# Single-stage versus staged interposition urethroplasty for glandular hypospadias with severe penile curvature: 15-year experience

**DOI:** 10.1007/s00345-021-03829-9

**Published:** 2021-09-05

**Authors:** Guanglun Zhou, Wanhua Xu, Jianchun Yin, Junjie Sun, Zhilin Yang, Shoulin Li

**Affiliations:** Department of Urology and Laboratory of Pelvic Floor Muscle Function, Shenzhen Children’s Hospital, Futian District, Shenzhen, 518000 Guangdong People’s Republic of China

**Keywords:** Penile curvature, Hypospadias, Urethroplasty, Interposition, Glandular

## Abstract

**Purpose:**

Our study examined the benefit of an alternative interposition urethroplasty (IU) procedure for glandular hypospadias (GH) with severe penile curvature (SPC). The technique involved transecting and reconstructing the urethra to preserve the distal glandular and coronal urethra and correct the curvature. We compared procedural characteristics, outcomes, and surgical complications for the single-stage and staged IU techniques.

**Methods:**

We retrospectively studied 44 patients with GH with SPC who underwent single-stage or staged IU between March 2005 and June 2020. Demographics, operative details, complications, and uroflometry findings were analyzed.

**Results:**

The median age at initial surgery was 37.5 months. Ten patients underwent single-stage IU repair, and 34 patients underwent staged IU repair. The median length of the interposition neourethra was 3.2 cm (2.2–4.3). The median follow-up duration was 58 months, and the overall complication rate was 13.6%. Complications were noted in 30% (3/10) and 8.8% (3/34) of patients in the single-stage and staged IU groups, respectively (*p* > 0.05). Fistula formation was noted in one and three patients in the single-stage and staged groups, respectively (8.8% vs. 10%, *p* > 0.05). Two cases of urethral stricture were documented in the single-stage group only. No chordee recurrence or urethral diverticula was noted in any of the patients.

**Conclusion:**

IU is a reliable and durable technique for GH with SPC. It avoided penile shortening, preserved the distal urethra, and reduced the risk of chordee recurrence. The staged IU technique had more superior outcomes compared to the single-stage IU technique.

## Introduction

Hypospadias is one of the most common congenital anomalies in children [1]. While most cases are distal hypospadias, they are rarely complicated by severe penile curvature (SPC) [2], because glandular hypospadias (GH) with SPC is an unusual deformity in children. Distal hypospadiases are usually repaired with a tubularized incised plate or the Mathieu or meatal advancement and glanuplasty inclusive techniques, but these are not suitable for GH with SPC [3, 4]. A few techniques have been proposed for this condition; however, none have been satisfactory [5]. Urethral division and reconstruction is often required to achieve successful repair, but there is still controversy as to whether (a) the distal urethra should be preserved or resected, (b) the ventral surface of the corpora should be lengthened or the dorsal surface shortened, and (c) single-staged or staged urethroplasty should be performed [5].

To our knowledge, there are limited studies on the treatment and outcomes of GH with SPC in children. Our study described our experience with an alternative interposition urethroplasty (IU) technique for GH with SPC in children and compared the efficiency and complications between single-stage and staged IU repair.

## Patients and methods

### Study design and population

This was a retrospective observational study. The study design was approved by the institutional review board of Shenzhen Children’s Hospital. Written informed consent was obtained from the parents or guardians of all patients prior to treatment.

**Fig. 1:**
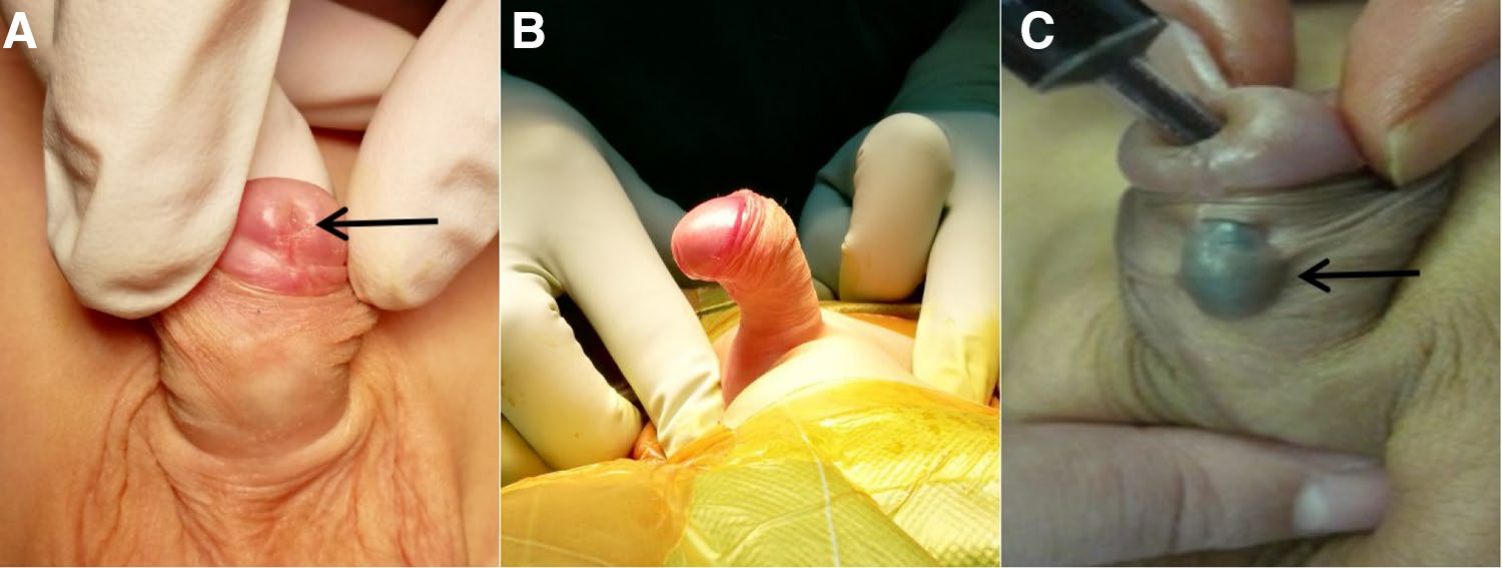
Preoperative images **A** glandular meatus; **B** severe penile curvature; **C** hypoplastic urethra with methylene blue injected through the meatus

We retrospectively examined all patients diagnosed with GH with SPC who underwent IU at our institution. GH with SPC was defined as a meatal location on the ventral side of the glans, curvature due to a short urethra, and curvature > 30° after degloving [2]. Cases may also have been accompanied by a hypoplastic urethra (Fig. 1A–C). In such cases, the distal urethra (glandular and coronal urethra) has a thickness that can be preserved. Twelve patients underwent single-stage IU between March 2005 and January 2011, whereas 34 patients underwent staged IU between February 2011 and June 2020. We converted to a staged procedure, because the single-stage procedure had complicated steps and several complications. Data on patient’s demographics, operative details, postoperative uroflometry, and complications were reviewed. Complications included urethrocutaneous fistula, urethral stricture, diverticulum, and residual chordee. Two patients from the single-stage IU group were excluded from the final analyses because of missing data. Patients with GH without chordee or with mild to moderate chordee were excluded.

### Surgical technique

#### Single-stage IU

We reassessed the penile curvature after complete penile degloving and excision of the tethering ventral tissue. If an SPC was present, the urethra was transected at the point of maximum ventral curvature to preserve the distal urethra. The transected urethra was then properly released at its two ends to straighten and lengthen the penis. An additional dorsal midline plication procedure was performed in patients in whom urethral transection was inadequate. Dorsal midline plication was performed by measuring the length of the remaining urethral defect and creating a neourethra, which was composed of a transverse preputial island flap wrapped around a catheter stent, to cover the urethral defect. The neourethra was transposed ventrally and anastomosed with the proximal and distal stoma of the transected urethra. The flap pedicle was then draped over the ventral aspect of the neourethra and sutured into place to cover the urethral suture line.

#### Staged IU

The first stage of staged IU, which comprised penile degloving, excision of the tethering ventral tissue, and urethral transection, was performed as described above. A Byars’ flap was then created and transferred to the ventral aspect of the penis to cover the urethral defect and create a new urethral plate.

**Fig. 2:**
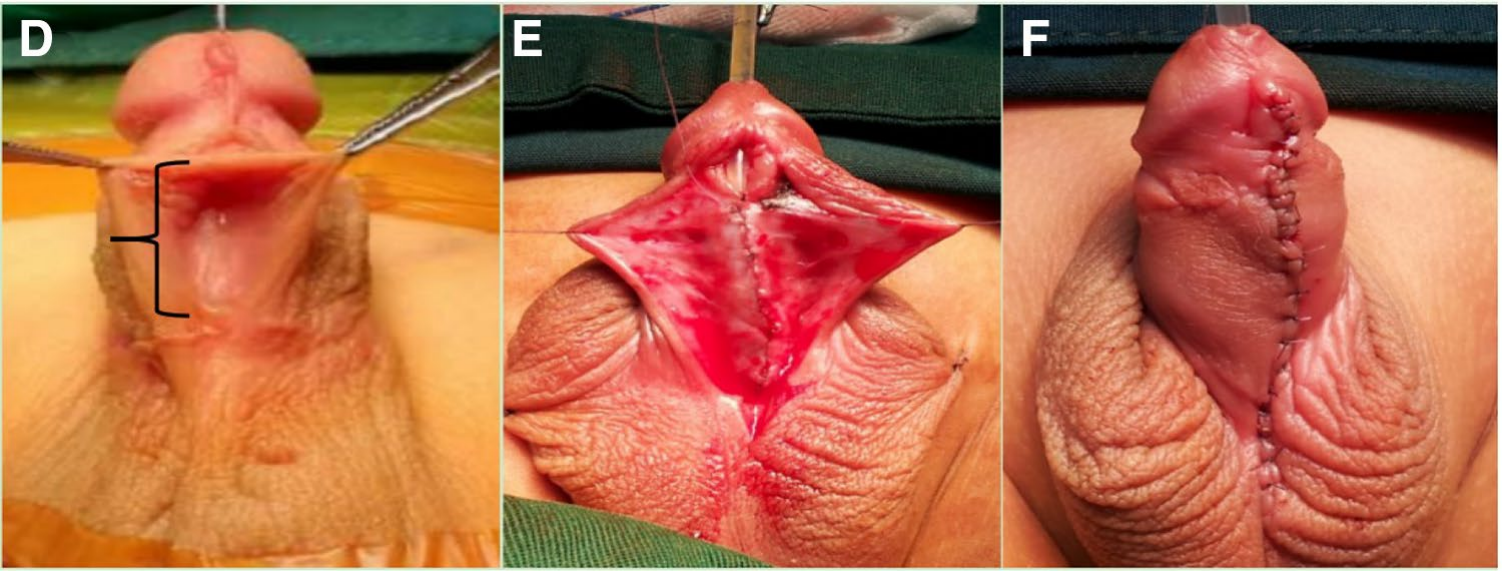
Intra-operative images of the second step of staged repair **D** length of the urethral defect (bracket). **E** Formation of the new urethral tube. **F** Postoperative appearance of the penis with preservation of the distal urethra

The second stage of the procedure was performed approximately six months later, and the Thiersch–Duplay technique was used to repair the urethral defects. We made a 12–15-mm incision along the ventral side of the penis that extended from the distal to proximal meatus (Fig. 2D). We dissected down to the Buck's fascia and performed tubularization using an 8- or 10-French silicone catheter (Fig. 2E). We closed the urethral plate with one or two layers of fine absorbable sutures. A second layer of closure was performed using a fascia and vessel pedicle flap. Ventral skin closure was performed using tensionless interrupted sutures (Fig. 2F).

### Follow-up

Patients were re-evaluated at 1, 6, and 12 months and annually thereafter. Uroflometry was performed during each follow-up.

### Statistical analysis

Statistical analysis was performed using SPSS version 22.0. Categorical variables were presented as numbers and percentages and compared using the Chi-squared test or Fisher’s exact test. Continuous variables that followed a normal distribution were described as means ± standard deviations and compared using the Student’s *t*-test. Statistical significance was set at *p* < 0.05.

## Results

We analyzed a total of 44 children; ten cases were treated with single-stage IU, and 34 cases were treated with staged IU. The demographic characteristics and outcomes of all patients and procedures, respectively, are summarized in Table 1. The median age at the initial surgery was 37.5 months. Seventeen cases had a hypoplastic urethra in the midshaft of the penis. There was no significant difference in the median length of the interpositional neourethra between the single-stage and staged IU groups (2.19 vs. 3.22, *p* = 0.84). In the staged IU group, the median time between both stages of repair was 8.2 months. A total of six cases underwent degloving + division of the plate followed by dorsal midline plication (two cases underwent simultaneous dorsal plication and ventral corporotomies).

**Table 1:** Demographic and clinical outcomes of the single-stage and staged IU groups

Variable	Single-stage	Staged	*P* value
Number of patients	10	34	
Age at first surgery (mo) (range)	37 (31–74)	39.8 (16–109)	0.63
Length of the urethral defect (cm) (range)	3.19 (2.2–4.2)	3.22 (2.5–4.3)	0.84
Complications	3 (30%)	3 (8.8%)	0.12
Fistula	1 (10%)	3 (8.8%)	1
Urethral stricture	2 (20%)	0 (0%)	0.04

The median follow-up time was 58 months, and the overall complication rate was 13.6% (6/44). In particular, we documented complications in 30% (3/10) and 8.8% (3/34) of the patients who underwent single-stage and staged IU, respectively (*p* = 0.12). The complications in the single-stage and staged IU groups included urethrocutaneous fistulas (10 and 8.8%, respectively). Two cases of urethral stricture were documented in the single-stage IU group only. All urethrocutaneous fistulas were successfully repaired with reoperations, and both cases of urethral strictures were treated successfully with dilatation. Postoperatively, all patients demonstrated a good penile cosmetic appearance with no chordee recurrence or urethral diverticula.

The single-stage IU group demonstrated lower maximal urinary flow rates at one month postoperatively compared to the staged IU group (6.4 ± 1.2 mL/s vs. 7.2 ± 1.0 mL/s, *P* < 0.05); however, there was no significant difference in the maximal urinary flow rates at 6 months and after 1 year between both groups (7.8 ± 1.6 mL/s vs. 8.3 ± 1.7 mL/s and 10.4 ± 2.2 mL/s vs. 10.6 ± 2.3 mL/s, respectively) (*P* > 0.05) (Table 2).

**Table 2:** Maximal urinary flow rates between the single-stage and staged groups

Variable	Single-stage	Staged	*P* value
*Maximal urinary flow rate (mL/s)*			
Three months after surgery	6.4 ± 1.2	7.2 ± 1.0	0.04
Six months after surgery	7.8 ± 1.6	8.3 ± 1.7	0.52
One year after surgery	10.4 ± 2.2	10.6 ± 2.3	0.94

## Discussion

Regardless of the surgical technique, the goals of surgery for GH with SPC is to create good functional and cosmetic outcomes with low complication rates [6]. The IU technique is an effective treatment for GH with SPC in children. Our results demonstrated that it provides high success rates and satisfactory functional outcomes at the 5-year follow-up.

The IU technique can restore the natural length of the penis and provide a viable, high-quality urethra for patients with GH with SPC. In the present study, patients with GH with SPC required treatment for chordee that developed due to a short urethra. After correcting the chordee, the location of the meatus was nearly normal and did not require further treatment. In other cases, the urethra may be preserved with dorsal plication of the tunica albuginea of the penis; however, excessive plication may lead to unacceptable penile shortening [7]. In our study, the median length of the reconstructed neourethra was 3.2 cm after correcting the chordee, which added to the overall length of the penis. Loss of penile length is a common dissatisfaction among patients who have undergone surgery to correct a chordee and is psychologically and physically debilitating for patients [8]. Greenfield et al*.* [8] considered penile shortening as a known complication after surgical correction for this disorder, whereas Moscardi et al*.*[9] reported that multiple dorsal plications of an SPC increases the risk for recurrence. Our study also demonstrated that 38.6% of patients had a hypoplastic urethra in the midshaft of the penis. Mobilization of the urethra was difficult in these patients, because the skin and urethral mucosa were densely adherent to each other [10]. Moreover, there was an increased risk for fistula formation during dissection of the hypoplastic urethra [5]. Therefore, reconstructing the urethra is the preferred choice for GH with SPC. We chose to transect the ventral urethra to fully correct the chordee and maximize penile length. Our technique also treated the hypoplastic urethra and provided a well-functioning neourethra for our patient.

The IU technique provided superior cosmetic results with low postoperative complications, because it preserved the glandular and coronal urethra. In the present study, we only reconstructed the midshaft of the penile urethra, which allowed us to retain the frenulum of the prepuce and slit-like meatus in the glans. Retaining the slit-like meatus is important in hypospadias repair [11], whereas the frenulum plays an important role in penile erection [12]. Preservation of the distal urethra also contributes to directing the urinary stream and limiting spraying. Robinson et al*.* [13] suggested that better cosmetic results lead to better sexual outcomes. The overall complication rate in our study was significantly lower than that reported by Sylvia et al. [14] (13.6% vs 50%). Tang et al. [15] treated patients by resecting the mid-distal segment of the penile urethra and reconstructing it with single-staged longitudinal island flap urethroplasty; they found that the urethroplasty had a 15.8% cumulative complication rate, a 5.3% fistula rate, a 5.3% stricture and a 5.3% residual curvature rate. Singh et al. [16] reported the single-staged tubularized transverse inner preputial island flap for the treatment of patients; its reoperation rate was 50%. However, the IU approach provided success rates of up to 86.4%, and the reoperation rate was only 9.9%. The approaches of Tang et al. [15] and Singh et al. [16] have some disadvantages: (a) a distal urethral reconstruction would have resulted in meatal stenosis and coronal fistula formation; (b) these procedures make postoperative coronal fistulas difficult to manage and are associated with an increased risk for meatal strictures; and (c) a slit-like urethral meatus is also difficult to create with these procedure.

Since 2005, 12 patients with GH with SPC underwent the single-stage IU technique; however, the results from the retrospective analysis of this technique were not satisfactory. In particular, single-stage repair of hypospadias had steep learning curves, and necrosis of the neourethra occurred due to poor flap blood supply [17]. In contrast, staged repair was simple. Since 2011, we exclusively performed staged repair in patients with GH with SPC in order to obtain better results and fewer complications.

Urethrocutaneous fistulas are a common complication of hypospadias [18]. The incidence of fistula formation in single-stage repair was lower than that in staged repair, but the difference was not statistically significant. In our study, all fistulas occurred in the distal body of the penis, but there was no glandular or coronal fistulas after the IU repair, because we preserved the distal urethra. Our results demonstrated a fistula repair success rate of 100%. We performed fistula repairs at least 6 months after urethroplasty and followed the basic principles of fistula repair [19].

In the present study, single-stage repair was significantly associated with complications compared to staged repair; two cases of urethral strictures were documented in the single-stage IU group, whereas none were noted in the staged repair group. Single-stage repair may be more prone to urethral strictures [20, 21], because it creates two circular anastomoses between the neourethra and original urethra, which increases the risk for neourethral tension or ischemia secondary to a transverse preputial island flap. On the other hand, the semi-circular anastomosis performed in the staged procedure prevents urethral strictures. Urethral stricture was mainly caused by a circular anastomosis between the native urethra and the neourethra in patients undergoing the single-stage procedure [22]. In first-stage repair, a semicircular anastomosis is created between the original urethra and the Byars’ flap. In second-stage repair, the Thiersch–Duplay procedure could decrease the urethral stricture rate due to avoid circular anastomosis [22]. Compared to fistulas, urinary strictures are more difficult to treat and require revision of the urethroplasty in severe cases [1]. In our study, patients with urethral strictures and urethral dilation were able to resume normal urination. Our urethral stricture rate was 4.5% and significantly lower than the 12.5% rate reported in a systematic review [20]. This is primarily because the majority of our patients underwent staged repair.

In our study, we chose to use Byars’ flap technique, instead of a free preputial graft, in the first stage of the staged repair because of the following reasons: (a) the Byars’ flap is stable and well-vascularized; (b) compared to the free preputial graft, the Byars’ flap technique has simpler steps; (c) the complication rates for a 2-stage repair using a free graft have a high incidence and the free graft may be completely lost [23]. Snodgrass [24] reported that the incidence of diverticulum in the Byars’ flap technique was higher than that of the free graft. However, no urethral diverticula was seen in this study, which may be due to the size of the Byars’ flaps created, which was consistent with the length of urethral defect.

In current study, most cases (86.4%) achieved complete penile straightening through degloving and division of the plate, which is similar to the results reported by Catti et al. [25]. Further treatment is necessary if mild curvature persists after transection of the urethral plate [26]. Dorsal plication appears to be the frequently used and effective approach [26]. Braga et al. [27] reported that no curvature recurrence was seen in patients with SPC undergoing simultaneous dorsal plication and urethral plate transection. Similarly, we used plication for cases of mild curvature persisting after degloving and division of the plate, which achieved good results. In our study, none of the patients had curvature recurrence, and the postoperative curvature assessments were based on photo documentation of the erect penis.

The present study assessed voiding function through uroflometry, which provided an objective index for measuring lower urinary tract function [13]. Our data demonstrated that despite the low maximal urinary flow observed at one year follow-up, the maximal urinary flow rate spontaneously and progressively improved in all patients at the 5-year follow-up. The improvement in maximal urinary flow rates may be due to support from the growth of the surrounding corpora and softening of the scar tissue in the neourethra [28]. Long-term follow-up with uroflometry is recommended after hypospadias repair, especially in cases with abnormal maximal urinary flow.

This study had some limitations. First, this was not a randomized controlled study, and it analyzed a retrospective series, thereby limiting our analyses. Second, the number of cases in the single-stage IU group was relatively small because surgeons converted the single-stage procedure to a staged procedure, and two cases were excluded because of missing data. Thus, only 10 patients were included in the single-stage group, which may have an impact on the results of the statistical analysis of the data. However, these cases are uncommon. Third, we lacked data on puberty outcomes. Our study population has to be followed up for over a longer time for these data to be evaluated. Fourth, the surgeries were performed by multiple surgeons, and variations in surgical techniques could have influenced the outcomes.

## Conclusions

We recommend the IU technique for the treatment of GH with SPC. Our findings suggested that an interpositional neourethra preserves penile length, reduces the risk for chordee recurrence, and provides superior cosmetic and functional results. Moreover, none of patients had curvature recurrence after the IU technique. Compared to single-stage IU repair, staged IU repair has simpler steps and a lower complication rate. The learning curve of staged IU is relatively short, and there are few serious complications. Residents training should be started with the simple hypospadias surgery, and then slowly carrying out this special type of staged procedure.

## Abbreviations

IU: Interposition urethroplasty
GH: Glandular hypospadias
SPC: Severe penile curvature
